# Complete Genome Sequences of Two Pasteurella multocida Isolates from Seabirds

**DOI:** 10.1128/mra.01365-22

**Published:** 2023-03-27

**Authors:** Amro Hashish, Timothy J. Johnson, Dhiraj Chundru, Michele L. Williams, Yuko Sato, Nubia R. Macedo, Augustin Clessin, Hubert Gantelet, Caroline Bost, Jérémy Tornos, Amandine Gamble, Karen J. LeCount, Mostafa Ghanem, Thierry Boulinier, Mohamed El-Gazzar

**Affiliations:** a Department of Veterinary Diagnostic and Production Animal Medicine, College of Veterinary Medicine, Iowa State University, Ames, Iowa, USA; b Department of Veterinary and Biomedical Sciences, College of Veterinary Medicine, University of Minnesota, Minneapolis, Minnesota, USA; c Department of Veterinary Medicine, Virginia-Maryland College of Veterinary Medicine, University of Maryland, College Park, Maryland, USA; d CEFE, University of Montpellier, CNRS, EPHE, IRD, Montpellier, France; e Ceva Biovac, Beaucouzé, France; f University of California, Los Angeles, California, USA; g National Veterinary Services Laboratories, United States Department of Agriculture, Ames, Iowa, USA; h National Laboratory for Veterinary Quality Control on Poultry Production, Animal Health Research Institute, Agriculture Research Center, Giza, Egypt; Indiana University, Bloomington

## Abstract

Pasteurella multocida is one of the major causes of mass mortalities in wild birds. Here, we report the complete genome sequences of two P. multocida isolates from wild populations of two endangered seabird species, the Indian yellow-nosed albatrosses (Thalassarche carteri) and the northern rockhopper penguins (Eudyptes moseleyi).

## ANNOUNCEMENT

Recurrent outbreaks of fowl cholera (FC), caused by Pasteurella multocida, have been associated with mass die-offs; threatening the population of nestling albatrosses on Amsterdam Island, in the southern Indian Ocean (1, 2, 15, 16, and 17). Dead birds were necropsied on this island as part of a wildlife health monitoring program conducted since 2013 (1, 3, 4). The fieldwork was approved by the French Regional Animal Experimentation Ethical Committee no. 036 (Ministry of Research permit 10257-2018011712301381) and by the Comité de l’Environnement Polaire (A-2018-123 and A-2018-139 for 2018 to 2019). During necropsy, femur bone marrow was swabbed, and swabs were incubated in liquid Amies transport medium before cultivation (up to 8 h). The swabs were then inoculated on Columbia sheep blood and incubated at room temperature (13 to 20°C) for up to 48 h. Most abundant type colonies were selected and stored in stock culture agar at room temperature during transportation to the laboratory (up to several months). They were then streaked on blood medium plates and incubated at 37°C. Characterization of the obtained clonal populations was performed by matrix-assisted laser desorption ionization time of flight mass spectrometry (MALDI-TOF MS) (5). Isolates identified as P. multocida were stored at −80°C until sequencing. Two isolates of P. multocida—one from an adult yellow-nosed albatross (Thalassarche carteri) and one from an adult northern rockhopper penguin (Eudyptes moseleyi)—were sequenced in this study (Table 1).

**TABLE 1:** Data associated with the two sequenced P. multocida strains

Type of data	Parameter^*a*^	Result for sequenced P. multocida strain	
		PM-32985	PM-33011
Metadata	Seabird species	Eudyptes moseleyi (adult)	Thalassarche carteri (adult)
	Geographical location	Amsterdam Island	Amsterdam Island
	Date of sample collection	30 October 2019	31 October 2019
	Organ of isolation	Femur bone marrow	Femur bone marrow

Raw sequence reads	Illumina paired-end read length (nt)	300	300
	No. of Illumina reads used	117,512	188,312
	Avg Illumina coverage, ×	15	23
	No. of Nanopore reads	12,004	20,863
	Avg Nanopore coverage, ×	72	104
	Nanopore read *N*_50_ (bp)	35,506	31,401

Assembly statistics for the closed genomes	No. of contigs	2	2
	Total length of the chromosome (bp)	2,428,887	2,443,848
	Total length of the plasmid (bp)^*b*^	3,741	3,741
	GC content (%)	40.33	40.29

Annotation results	No. of CDSs (with protein)	2,270	2,275
	No. of tRNAs	57	58
	No. of rRNAs	19	19
	No. of ncRNAs	4	4
	No. of CDSs (without protein)	34	14
	No. of CRISPR arrays	3	3
	No. of hypothetical proteins	169	178
	No. of proteins with functional assignments	2,101	2,097

MLST	ST by RIRDC scheme^*c*^	ST61	ST61
	ST by multiple-host scheme^*d*^	ST91	ST91
DNA fingerprinting		1,710	1,710
Serotyping	AGID	1, 14	1, 14
ANI (%)^*e*^	Identity to P. multocida type strain NCTC10322 (LT906458.1)	98.62	98.63

GenBank data	BioSample accession no.	SAMN28552975	SAMN28552996
	BioProject accession no.	PRJNA839711	PRJNA839711
	Complete genome assembly accession no.	Chromosome: CP097610	Chromosome: CP097612
		Plasmid: CP097611	Plasmid: CP097613

a CDSs, coding DNA sequences; ncRNAs, noncoding RNAs; MLST, multilocus sequence typing; RIRDC, Rural Industries Research and Development Corporation; AGID, agar gel immune diffusion.

b The presence or absence of plasmids in the genome sequences was determined using the plasmid database (11) available via PLSDB at https://ccb-microbe.cs.uni-saarland.de/plsdb.

c MLST was performed using the P. multocida RIRDC MLST scheme (12) available via PubMLST (13) at https://pubmlst.org/organisms/pasteurella-multocida.

d MLST was performed using the P. multocida multiple-host MLST scheme available via PubMLST (13).

e The percentage of average nucleotide identity (ANI) was calculated based on BLAST+ (14) available via the JSpeciesWS online service at https://jspecies.ribohost.com/jspeciesws/#home.

Selected isolates were sequenced with the Illumina and Nanopore sequencing platforms for the purpose of hybrid assembly. The Circulomics Nanobind CBB Big DNA kit (Circulomics, Baltimore, MD, USA) was used for extraction of high-molecular-weight DNA for Nanopore sequencing. Nanopore libraries were prepared using the SQK-LSK109 and EXP-NBD104 kits according to the manufacturer’s protocol (Oxford Nanopore Technologies, United Kingdom). DNA shearing and size selection were not performed. Sequencing libraries were sequenced using MinION Mk1B sequencer (Oxford Nanopore Technologies) with R9.4.1 flow cells. For Illumina sequencing, bacterial DNA extraction was performed using MagMax pathogen RNA/DNA kit (Thermo Fisher Scientific, Waltham, MA, USA), as previously described (6), on a Kingfisher-Flex instrument (Thermo Fisher Scientific, Waltham, MA, USA). Subsequently, extracted DNA was used for the preparation of sequencing libraries using Illumina DNA Prep tagmentation kit (previously called Nextera DNA Flex) with IDT for Illumina DNA/RNA UD indexes and set A according to the manufacturer’s protocol (Illumina, USA). Sequencing was performed using the Illumina MiSeq system (v3 reagent kit using 2 × 300-bp paired-end reads).

Raw reads from Illumina data were trimmed for quality, and sequencing adapters were removed using Trimmomatic v.0.33 (7). For Nanopore sequencing, raw Nanopore electrical signal (.fast5) was converted to base sequence (.fastq) through high-accuracy basecalling using Guppy (v.4.2.3) within MinKNOW (20.10.6) software. NanoFilt version 2.8.0 was then used to quality-filter Nanopore reads and to filter out sequences less than 1,000 bp in length (8). Hybrid assemblies were performed using Unicycler v.0.5.0 (9). Default parameters were used for the software mentioned. Each of the sequenced genomes showed 2 circular contigs (1 chromosome and 1 plasmid). The genomes were annotated using NCBI Prokaryotic Genome Annotation Pipeline (PGAP) v.6.1 (10).

Sequencing, annotation, and typing results for the sequenced isolates are presented in Table 1. The availability of these complete genomes will help us to understand the source of P. multocida infections among seabirds through comparison to P. multocida genomes from other species.

### Data availability.

The genomes are available from NCBI BioProject no, PRJNA839711 under the complete genome assembly accession no. (CP097610, CP097611, CP097612, and CP097613) shown in Table 1.
